# Adipocytes cause leukemia cell resistance to daunorubicin via oxidative stress response

**DOI:** 10.18632/oncotarget.12246

**Published:** 2016-09-26

**Authors:** Xia Sheng, Jonathan Tucci, Jean-Hugues Parmentier, Lingyun Ji, James W. Behan, Nora Heisterkamp, Steven D. Mittelman

**Affiliations:** ^1^ Diabetes and Obesity Program, Center for Endocrinology, Diabetes and Metabolism, Children's Hospital Los Angeles, Los Angeles, CA, USA; ^2^ Department of Biostatistics, Children's Hospital Los Angeles, Los Angeles, CA, USA; ^3^ Division of Hematology/Oncology and Bone Marrow Transplant, Children's Hospital Los Angeles, Los Angeles, CA, USA; ^4^ Department of Pediatrics, Keck School of Medicine, University of Southern California, Los Angeles, CA, USA; ^5^ Department of Pathology, Keck School of Medicine, University of Southern California, Los Angeles, CA, USA; ^6^ Departments of Physiology and Biophysics, Keck School of Medicine, University of Southern California, Los Angeles, CA, USA

**Keywords:** ALL, adipocyte, oxidative stress, glutathione, drug resistance

## Abstract

Adipocytes promote cancer progression and impair treatment, and have been shown to protect acute lymphoblastic leukemia (ALL) cells from chemotherapies. Here we investigate whether this protection is mediated by changes in oxidative stress. Co-culture experiments showed that adipocytes protect ALL cells from oxidative stress induced by drugs or irradiation. We demonstrated that ALL cells induce intracellular ROS and an oxidative stress response in adipocytes. This adipocyte oxidative stress response leads to the secretion of soluble factors which protect ALL cells from daunorubicin (DNR). Collectively, our investigation shows that ALL cells elicit an oxidative stress response in adipocytes, leading to adipocyte protection of ALL cells against DNR.

## INTRODUCTION

Excess body weight contributes to as many as 1 out of 5 of all cancer-related deaths [1]. A landmark paper by Calle et al. found a significant association between obesity and mortality from many types of cancer [2]. Many solid tumors reside in locations rich with adipocytes, including breast, ovarian, pancreatic, colon, and prostate cancers, and some studies have shown that adipocytes interact with cancer cells [3–6]. Obesity has also shown to be associated with poorer event-free survival in the hematological cancer, acute lymphoblastic leukemia (ALL) [7–9], and we and others have demonstrated that leukemia/lymphoma cells can be found in close proximity to adipocytes in the bone marrow [10–12] and adipose tissue [13–15]. We also showed that adipocytes protect ALL cells from a variety of chemotherapeutic agents, though the mechanisms of this protection remain unclear [16, 17]. Here, we investigate the role of adipocytes in alleviating oxidative stress caused by chemotherapy treatment in ALL cells.

Cancer cells contain increased intracellular reactive oxygen species (ROS) due to metabolic and signaling aberrations [18, 19]. This increased ROS can have both beneficial and detrimental effects [20, 21]. Cancer cells maintain a balance of ROS by activation of oxidative stress response genes, many of which are regulated by the transcription factor nuclear factor erythroid 2-related factor 2 (Nrf2) [22, 23]. The increased expression of oxidative stress response genes contributes to cancer cell resistance to chemotherapies such as anthracyclines [24, 25], which work in part by inducing oxidative stress. Given the significant protection that adipocytes provide to ALL cells, particularly against the anthracycline daunorubicin (DNR) [16], we investigated whether adipocytes protect ALL cells from chemotherapies by relieving oxidative stress.

## RESULTS

### Adipocytes protect ALL cells from oxidative stress-induced cell death

**Figure 1:**
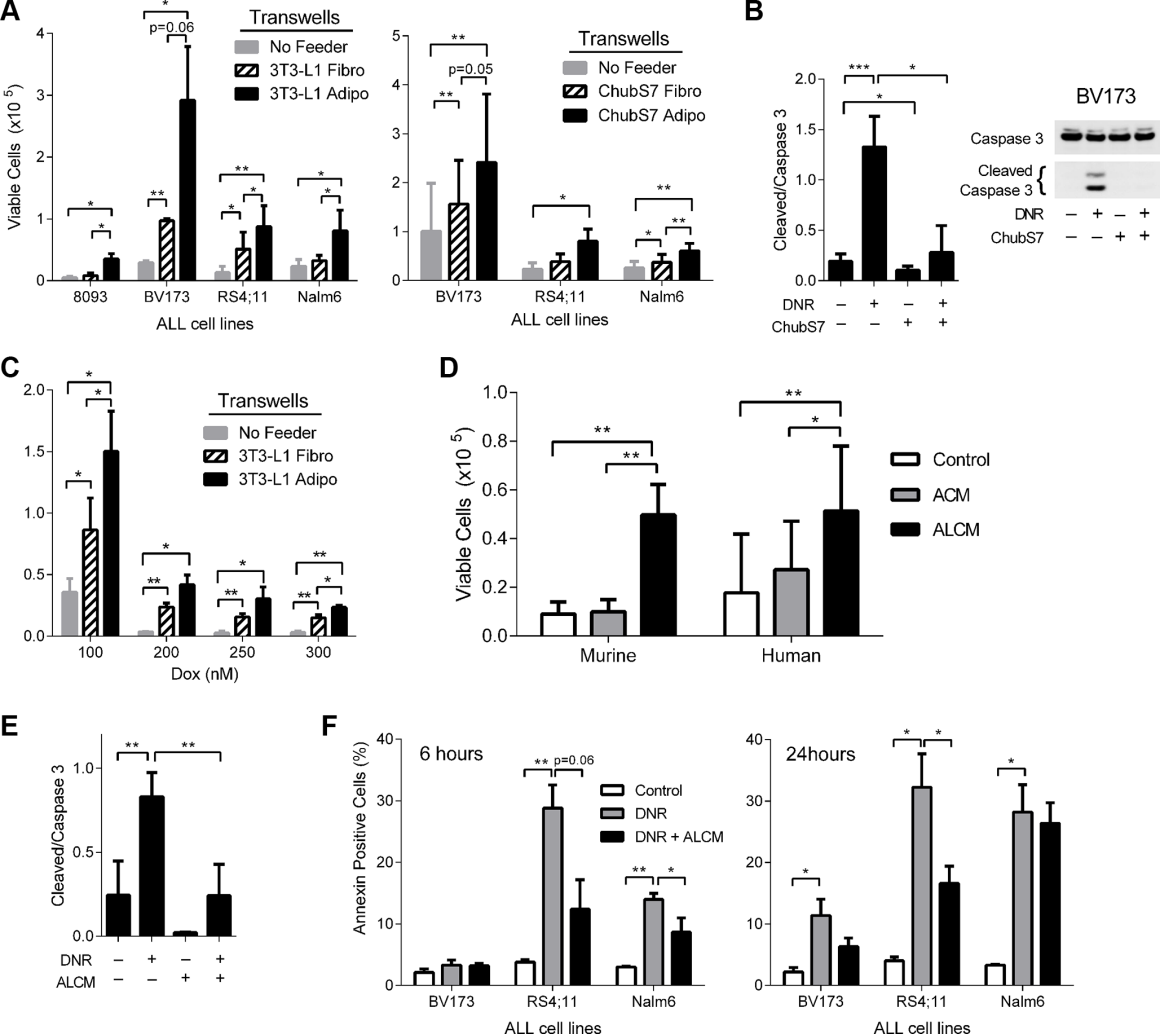
Adipocytes protect ALL from oxidative stress induced cell death (**A**) ALL cells co-cultured in TransWells over 3T3-L1 and ChubS7 pre-adipocytes (hatched bars) and adipocytes (black bars) with 72 hour DNR treatment (8093 35 nM, BV173 100 nM, RS4;11 100 nM, and Nalm6 200 nM). No feeder condition is where ALL cells were cultured alone (*n* = 3–5). (**B**) One representative image of western blot of Caspase 3 (37 kDa) and cleaved caspase 3 (19 and 17 kDa) in BV173 cells treated with DNR for 24 hours in the presence or absence of adipocytes. Images were histogram stretched in a consistent manner to increase brightness for publication. Quantification of the ratio of cleaved (both bands) over total caspase 3 (band at 37 kDa) is shown on the right (*n* = 3). (**C**) BV173 ALL cells cultured over 3T3-L1 cells and treated with various doses of doxorubicin (Dox). (**D**) 8093 cells treated with 35 nM DNR in 3T3-L1 ACM and ALCM (left, *n* = 6); BV173 cells treated with 100 nM DNR in ChubS7 ACM and ALCM (right, *n* = 5). (**E**) Quantification of cleaved over total caspase 3 of 8093 ALL cells after 24 hours treatment with 25 nM DNR with and without ALCM. (**F**) Annexin V staining of human ALL cells after 24 hour exposure to DNR with or without ALCM. *P < 0.05, **P < 0.01, ***P < 0.001 All asterisks indicate comparison to control no feeder (NF gray bar) or no DNR no adipocyte conditions (white bar) unless otherwise indicated.

We have previously reported that murine 3T3-L1 adipocytes protect murine ALL cells (8093) [26] from DNR in a direct co-culture system [16]; however, we did not test whether protection against DNR requires cell-cell contact, nor did we test whether this occurs with human ALL cell lines. Therefore, we cultured ~2 × 10^5^ mouse (8093) and human (BV173, RS4;11, and Nalm6) ALL cells in TransWells above 3T3-L1 adipocytes, preadipocytes (fibroblasts), or no feeder layer. Transwells contained polycarbonate membranes with 0.4 μm pores, allowing free diffusion of media without direct cell-cell contact. Adipocytes protected mouse and human ALL cells from DNR, compared to no feeder and pre-adipocytes (Figure 1A). The human adipocyte Chub-S7 cell line also protected human ALL cells against DNR in similar transwell cultures. While 24 hours of DNR treatment led to increased cleaved caspase 3 in BV173 cells, indicating apoptosis by the intrinsic pathway, this was reversed in the presence of adipocytes (Figure 1B). 3T3-L1 adipocytes also protected BV173 ALL cells from the anthracycline doxorubicin (Figure 1C).

Our data indicates that direct cell-cell contact is not necessary for the protection against DNR, implying the existence of soluble factors mediating this protection. Surprisingly, we found that adipocyte conditioned media (ACM) did not protect ALL cells from DNR (Figure 1D). However, media conditioned by both adipocytes and leukemia cells simultaneously (ALCM) protected 8093 and BV173 from DNR treatment compared to both control media and ACM (Figure 1D), and reduced DNR induced cleavage of caspase 3 in 8093 cells (Figure 1E). Similar results were observed when media was conditioned serially by ALL cells and then adipocytes (not shown). ALCM also protected ALL cells from apoptosis as measured by Annexin V, though this did not reach statistical significance in BV173 cells (Figure 1F). Together, these results imply that adipocytes release protective factors when stimulated by ALL cells.

**Figure 2:**
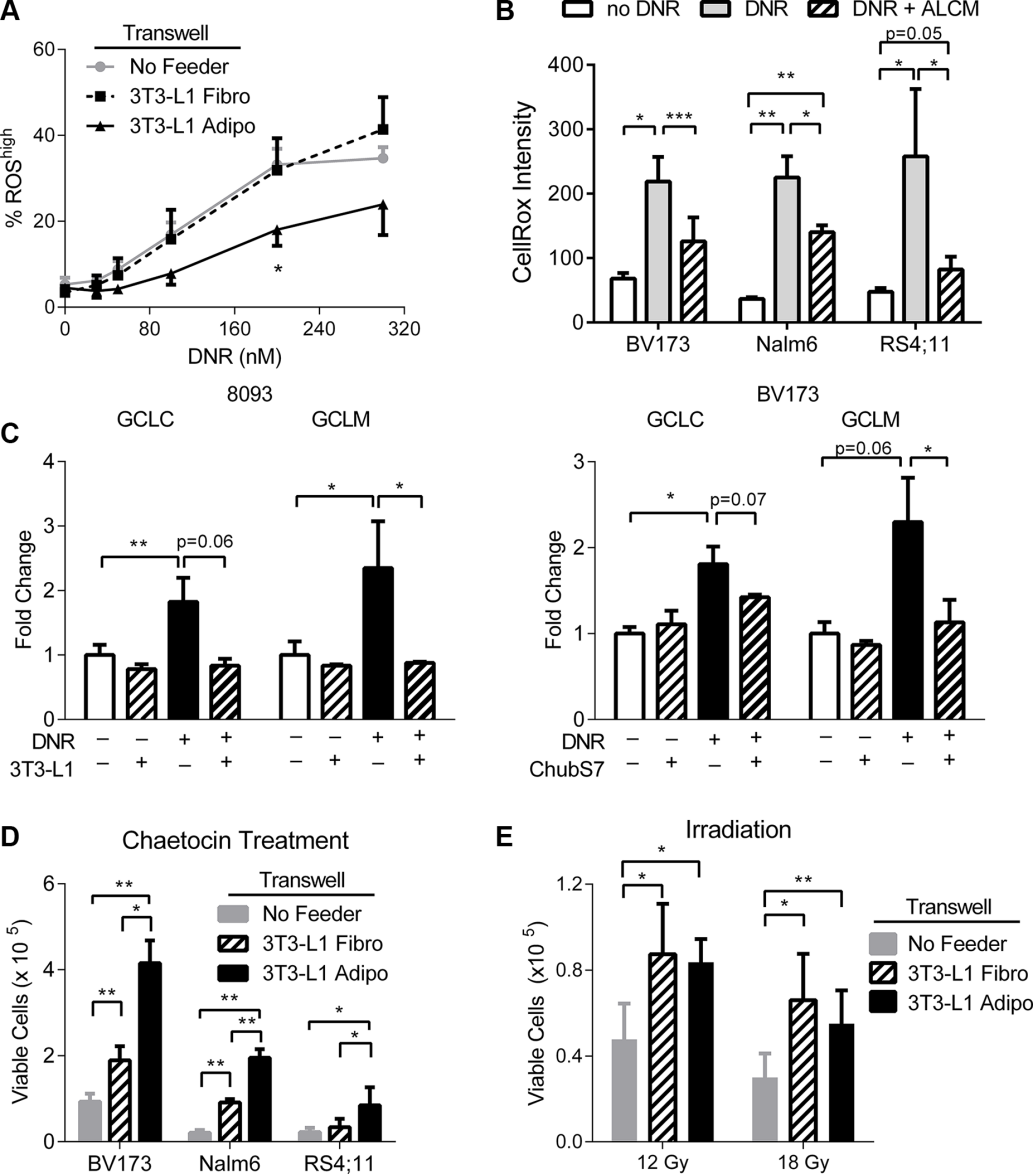
Adipocytes protect ALL cells from oxidative stress (**A**) Measurement of % ROS^high^ population in Nalm6 cells treated with DNR for 6 hours. % ROS^high^ based on gates defined by cells without DNR treatment. Adipo condition is significantly different than both Fibro and No Feeder by repeated measure ANOVA (*p* < 0.05). (**B**) CellRox^®^ intensity by flow cytometry measures intracellular ROS after 24 hours exposure to DNR or DNR plus ALCM. (**C**) GCLC and GCLM gene expressions by qPCR in 8093 and BV173 cells treated with DNR, with or without adipocytes present (*n* = 3). Asterisks and *p*-values indicate comparisons to the no-daunorubicin, no 3T3-L1 condition (white bar), unless otherwise noted. (**D**) ALL cells co-cultured with 3T3-L1 pre-adipocytes and adipocytes with chaetocin treatment (BV173 and Nalm6 100 nM, RS4;11 50 nM, *n* = 3–4). (**E**) Irradiated BV173 ALL cells co-cultured with 3T3-L1 pre-adipocytes and adipocytes (*n* = 3). *P < 0.05, **P < 0.01, ***P < 0.001.

Since DNR is a potent inducer of ROS [27], which contributes to its cytotoxicity, we next investigated whether adipocytes prevented DNR induction of ROS. We used DCFH-DA to directly measure intracellular ROS in Nalm6 cells during DNR treatment. DNR induced intracellular ROS within 6 hours in a dose-dependent manner, and this was significantly reduced in the presence of adipocytes, but not fibroblasts (Figure 2A). A similar pattern was observed in using the dye CellRox^®^, which demonstrated that ALCM protected all three human ALL cell lines from the DNR induction of intracellular ROS (Figure 2B). ALL cells exhibited an oxidative stress response after DNR treatment, as demonstrated by an increase in gene expression of the two subunits of the glutathione producing enzyme, glutamate cysteine ligase (GCLC and GCLM, Figure 2C). However, co-culturing with either 3T3-L1 or Chub-S7 adipocytes reversed the induction of these oxidative stress response genes (Figure 2C). Interestingly, expression of both genes decreased slightly, but significantly, in 8093 cells co-cultured with adipocytes without DNR treatment (black bar vs. white bar), potentially indicating alleviation of the basal level of oxidative stress. A similar pattern of GCLC and GCLM expression was also observed with Nalm6 ALL cells (data not shown).

To test whether adipocyte protection of ALL cells from oxidative stress was specific to that induced by DNR, we used additional inducers of oxidative stress. ALL cells were treated with chaetocin, a fungal-derived toxin that inhibits thioredoxin reductase-1, leading to increased cellular oxidative stress. Adipocytes significantly protected all three ALL cell lines from chaetocin induced cell death (Figure 2D). We also found that co-culturing with adipocytes modestly improved the viability of BV173 ALL cells that had been irradiated with 12 and 18 Gy (Figure 2E), though in this case adipocytes did not offer significantly more protection than pre-adipocytes. Although both chaetocin and irradiation cause insults besides just oxidative stress, these results are consistent with the findings that adipocytes protect ALL cells from oxidative stress induced cytotoxicity.

### ALL cells induce oxidative stress in adipocytes

**Figure 3:**
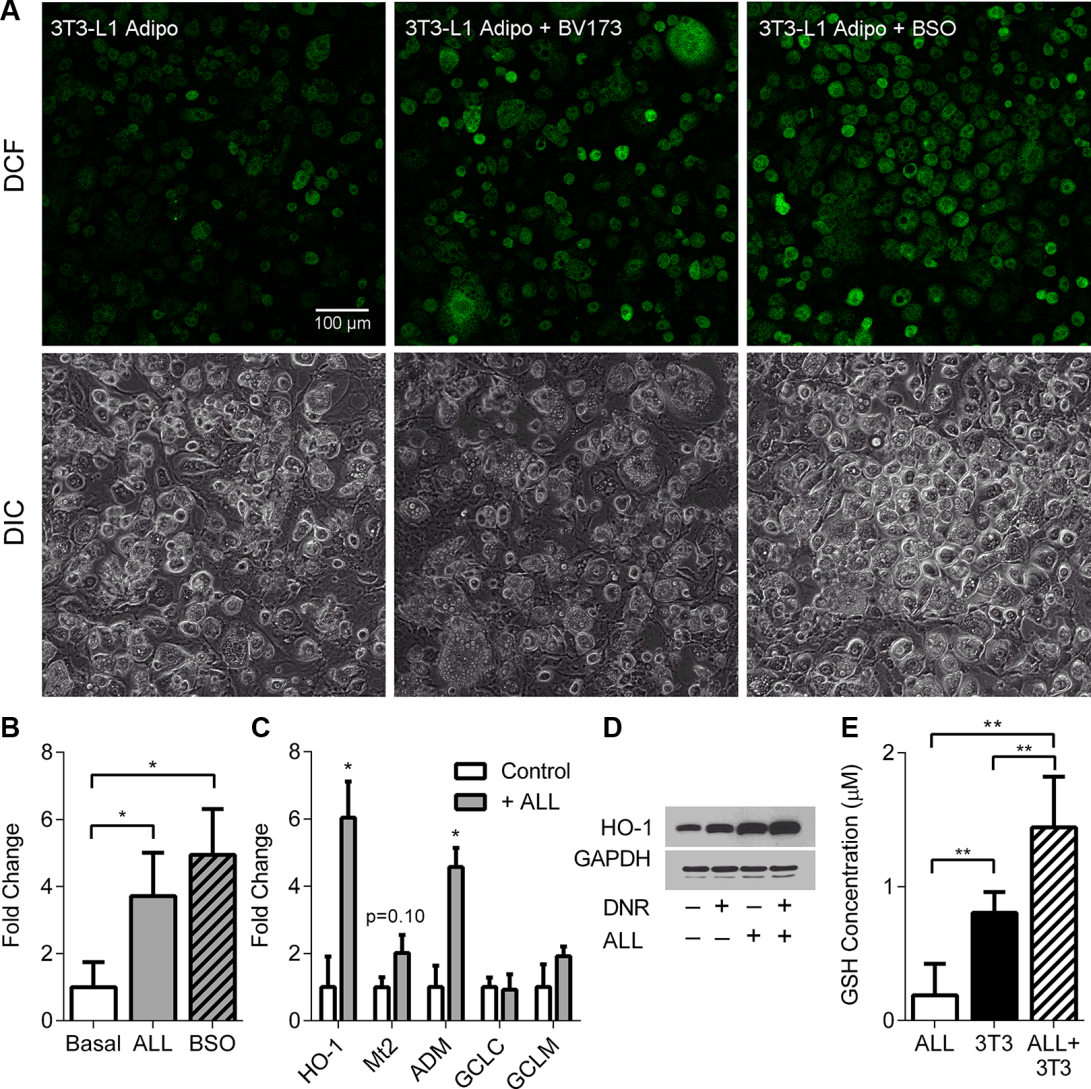
ALL cells induce oxidative stress in adipocytes (**A**) Representative fluorescent confocal microscopy images of 3T3-L1 adipocytes alone, with ALL in TransWells, or treated with 20 mM BSO for 48 hours. Top: DCF (green) only, bottom: DIC images. (**B**) Quantification of fluorescence (FITC) from 3 images similar to A. *indicate *t*-tests on log-transformed pixel counts. (**C**) 3T3-L1 gene expression by qPCR with (gray bar) and without (white bar) 8093 cells; *n* = 3. (**D**) Western blot of HO-1 expression in 3T3-L1 adipocytes exposed to DNR, ALL, or both. GAPDH was used as loading control. E. GSH levels measured in media after conditioning with BV173 ALL cells, 3T3-L1 adipocytes, or both, for 48 hours. *P < 0.05, **P < 0.01, ***P < 0.001.

To investigate how ALL cells stimulate adipocytes to secrete protective factors, we exposed adipocytes to leukemia cell conditioned media (LCM) for 24 hours, and performed Affymetrix microarray analysis on the adipocytes. We found 3,528 significant gene expression changes (*p* < 0.05) in the adipocytes, including upregulation of many genes in the oxidative stress response pathway (Table 1 shows the top 10 most upregulated genes, ranked by fold change). Ingenuity Pathway Analysis (IPA) identified the Nrf2-mediated oxidative stress response to be one of the most affected pathways in adipocytes, with 47 genes differentially regulated down-stream of the pathway (Ingenuity^®^ Systems, www.ingenuity.com, Table 2), implying that ALL cells were inducing oxidative stress in adipocytes. To directly test whether ALL cells induce oxidative stress in adipocytes, we visualized adipocyte intracellular ROS with DCFH-DA using fluorescence confocal microscopy. After 48 hours of co-culture with BV173 cells in a TransWell system, adipocytes had increased intracellular ROS (Figure 3A and 3B), to a similar degree as adipocytes treated with buthionine sulfoximine (BSO) as a positive control. Co-culture with ALL cells also induced a significant upregulation of the oxidative stress response genes, HO-1 and ADM, and a non-statistically significant increase in Mt2 gene expression (Figure 3C), as well as increased HO-1 protein levels (Figure 3D), confirming our microarray findings. Consistent with this, glutathione concentration in media conditioned by 3T3-L1 adipocytes together with BV173 ALL cells for 48 hours was higher than media conditioned by adipocytes or leukemia cells alone (Figure 3E).

**Table 1:** Top 10 most upregulated genes ranked by fold change from Affymetrix microarray analysis of data comparing gene expression changes in 3T3-L1 adipocytes exposed to LCM for 24 hours to control 3T3-L1 adipocytes

Gene symbol	Name	Fold change	*P*-value
Mt2	Metallothionein 2	4.6	0.02
Aldh1l2	Aldehyde dehydrogenase 1 family, member l2	3.6	0.00004
Adm	Adrenomedullin	3.5	0.0006
Trib3	Induced in fatty liver dystrophy 2	3.1	0.002
Eno2	Enolase 2, gamma neuronal	3.0	0.00002
Hmox1	Heme oxygenase (decycling) 1	2.7	0.0001
Ddit3	Dna-damage inducible transcript 3	2.7	0.0003
Myd116	Myeloid differentiation primary response gene 116	2.6	0.0003
Car6	Carbonic anhydrase 6	2.5	0.004
Got1	Glutamate oxaloacetate transaminase 1, soluble	2.5	0.003

**Table 2:** Forty-seven differentially regulated genes in the Nrf-2 mediated oxidative stress response pathway identified by IPA

Symbol	Entrez gene name	Fold change	*p*-value
HMOX1	heme oxygenase (decycling) 1	2.738	1.30E-04
GCLC	glutamate-cysteine ligase, catalytic subunit	2.020	2.40E-03
ABCC1	ATP-binding cassette, sub-family C (CFTR/MRP), member 1	2.007	1.63E-03
NQO1	NAD(P)H dehydrogenase, quinone 1	1.999	3.85E-03
FMO1	flavin containing monooxygenase 1	−1.779	2.97E-03
ATF4	activating transcription factor 4	1.740	4.28E-04
PIK3R3	phosphoinositide-3-kinase, regulatory subunit 3 (gamma)	−1.734	6.34E-03
MAFF	v-maf avian musculoaponeurotic fibrosarcoma oncogene homolog F	1.618	1.54E-02
MGST3	microsomal glutathione S-transferase 3	−1.569	5.49E-03
GCLM	glutamate-cysteine ligase, modifier subunit	1.541	1.19E-03
EPHX1	epoxide hydrolase 1, microsomal (xenobiotic)	1.502	9.86E-04
ACTG2	actin, gamma 2, smooth muscle, enteric	−1.493	2.22E-02
HERPUD1	homocysteine-inducible, endoplasmic reticulum stress-inducible, ubiquitin-like domain member 1	1.484	7.61E-03
MAP3K1	mitogen-activated protein kinase kinase kinase 1, E3 ubiquitin protein ligase	1.477	3.31E-03
ACTA2	actin, alpha 2, smooth muscle, aorta	−1.440	1.53E-02
SQSTM1	sequestosome 1	1.435	8.34E-04
MAFG	v-maf avian musculoaponeurotic fibrosarcoma oncogene homolog G	1.431	3.91E-02
PRKCD	protein kinase C, delta	1.428	3.99E-03
TXNRD1	thioredoxin reductase 1	1.414	2.83E-03
HSPB8	heat shock 22kDa protein 8	1.413	3.38E-04
MAP2K1	mitogen-activated protein kinase kinase 1	1.408	1.56E-04
PIK3R1	phosphoinositide-3-kinase, regulatory subunit 1 (alpha)	−1.406	1.68E-02
DNAJA3	DnaJ (Hsp40) homolog, subfamily A, member 3	1.398	3.47E-03
SCARB1	scavenger receptor class B, member 1	−1.374	2.06E-02
GSTO1	glutathione S-transferase omega 1	1.362	2.05E-02
DNAJC15	DnaJ (Hsp40) homolog, subfamily C, member 15	−1.301	3.46E-02
MAPK7	mitogen-activated protein kinase 7	1.284	1.06E-02
CAT	catalase	−1.271	1.84E-02
DNAJB2	DnaJ (Hsp40) homolog, subfamily B, member 2	1.262	2.98E-02
DNAJC16	DnaJ (Hsp40) homolog, subfamily C, member 16	1.247	2.02E-02
DNAJC8	DnaJ (Hsp40) homolog, subfamily C, member 8	1.228	1.41E-02
ENC1	ectodermal-neural cortex 1 (with BTB domain)	−1.203	1.06E-02
DNAJC18	DnaJ (Hsp40) homolog, subfamily C, member 18	1.203	2.90E-02
GSTM2	glutathione S-transferase mu 2 (muscle)	−1.199	2.73E-02
FTH1	ferritin, heavy polypeptide 1	1.195	3.25E-02
PIK3CA	phosphatidylinositol-4,5-bisphosphate 3-kinase, catalytic subunit alpha	1.190	5.38E-03
AOX1	aldehyde oxidase 1	−1.180	8.24E-03
DNAJC5	DnaJ (Hsp40) homolog, subfamily C, member 5	1.170	4.87E-02
JUN	jun proto-oncogene	1.148	1.33E-02
GSTO2	glutathione S-transferase omega 2	−1.147	4.86E-03
STIP1	stress-induced phosphoprotein 1	1.144	2.21E-02
EIF2AK3	eukaryotic translation initiation factor 2-alpha kinase 3	1.138	4.41E-02
PRKD1	protein kinase D1	−1.132	1.81E-02
MAPK3	mitogen-activated protein kinase 3	1.132	7.71E-05
FOSL1	FOS-like antigen 1	−1.110	1.41E-02
PIK3C3	phosphatidylinositol 3-kinase, catalytic subunit type 3	1.107	1.44E-02
DNAJC11	DnaJ (Hsp40) homolog, subfamily C, member 11	−1.069	4.86E-02

### ALL cell induction of oxidative stress in adipocytes induces their protection of ALL cells from DNR

**Figure 4:**
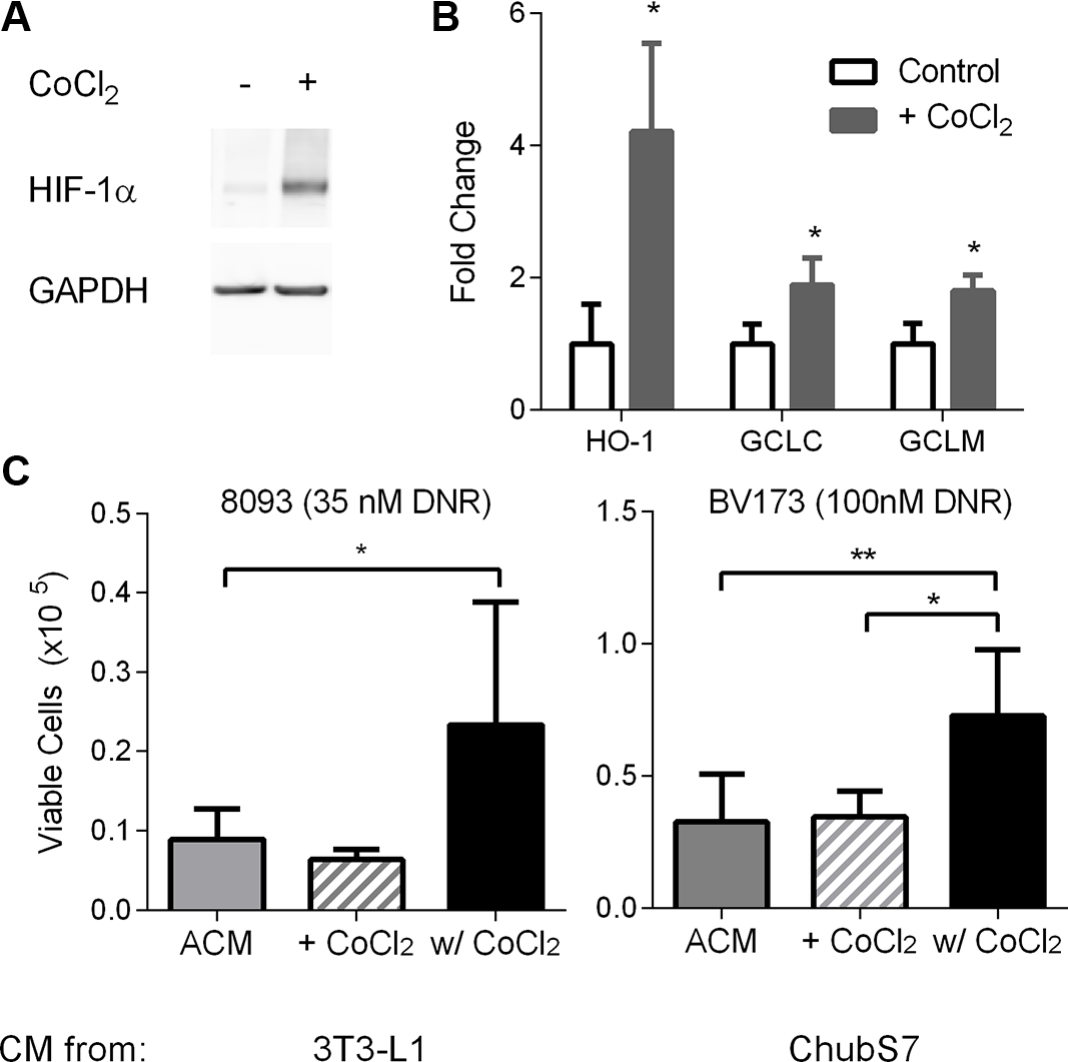
Oxidative stress in adipocytes leads to secretion of survival factors that protect ALL cells from DNR (**A**) Representative western blot of HIF-1α in ChubS7 adipocytes treated with CoCl_2_. GAPDH was used as loading control. (**B**) 3T3-L1 gene expression by qPCR with CoCl_2_ treatment for 24 hours (*n* = 4). (**C**) Viable cell number of DNR-treated 8093 ALL cells (left) or BV173 cells (right) in the presence of ACM from 3T3-L1 or ChubS7. ACM spiked with CoCl_2_ were denoted as (+ CoCl_2_) and ACM conditioned in the presence of 30 μM CoCl_2_ as (w/ CoCl_2_, *n* = 4–8). *P < 0.05 All asterisks indicate comparison to control conditions unless otherwise indicated.

To test whether ALL induction of oxidative stress response in adipocytes induces their protection of ALL cells, we treated adipocytes with cobolt chloride, a hypoxia mimetic which stabilizes HIF1α and induces an antioxidant response [28]. CoCl_2_ treatment was associated with increase in adipocyte HIF-1α protein levels and gene expression of HO-1, GCLC, and GCLM (Figure 4A and 4B). 3T3-L1 and ChubS7 conditioned media made in the presence of 30μM cobalt chloride (w/ CoCl_2_) were protective of ALL cells against DNR, compared to both ACM (Figure 4C), and ACM to which CoCl_2_ was added after conditioning as a control (+ CoCl_2_). Thus, induction of oxidative stress response by CoCl_2_ induces adipocytes to secrete factors which protect ALL cells from DNR.

**Figure 5:**
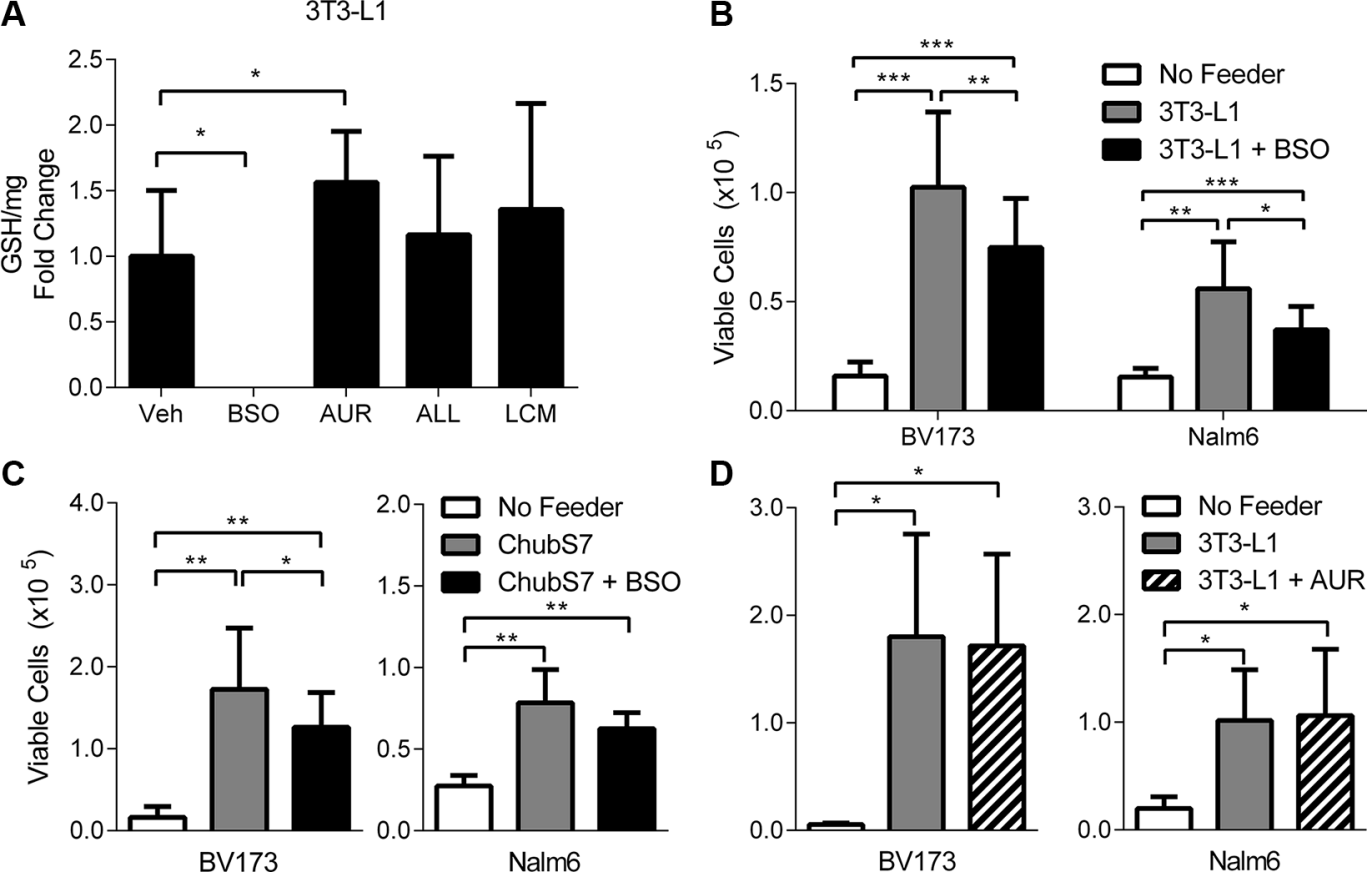
Glutathione synthesis is partially involved in adipocyte protection of ALL cells (**A**) Intracellular GSH quantification in 3T3-L1 adipocytes (*n* = 3). Viable cell number of BV173 and Nalm6 cells treated with DNR (100 and 200 nM, respectively) while co-cultured with 3T3-L1 (**B**) or ChubS7 (**C**) adipocytes that were pre-treated with 20 mM BSO for 24 hours (*n* = 8–10). (**D**) Viable cell number of BV173 and Nalm6 cells treated with DNR while co-cultured with 3T3-L1 adipocytes that were pre-treated with 250 nM AUR for 24 hours (*n* = 3).*p < 0.05, **p < 0.01, ***p < 0.001 All asterisks indicate comparison to no feeder or vehicle (Veh) conditions unless otherwise indicated.

To further hone in on which aspects of the adipocyte oxidative stress response are responsible for their protection of ALL cells against DNR, we blocked parts of the oxidative stress response in adipocytes prior to coculturing with ALL cells. BSO, an inhibitor of glutamylcysteine ligase, completely suppressed intracellular GSH levels in adipocytes, while auranofin (AUR), an inhibitor of thioredoxin reductase, actually increased GSH levels, likely a compensatory effect from blocking thioredoxin synthesis (Figure 5A). Neither treatment resulted in visible morphological changes to the adipocytes. ALL cells and LCM had no measurable effect on adipocyte GSH levels. Pretreatment of 3T3-L1 adipocytes with BSO only partially reversed their ability to protect BV173 and Nalm6 against DNR (Figure 5B). Similar results were observed with ChubS7 human adipocytes (Figure 5C). However, pretreatment of 3T3-L1 adipocytes with AUR did not affect DNR protection (Figure 5D), nor did it add to the effect of BSO to reduce adipocyte protection (not shown). Taken together, these results demonstrate that GSH synthesis may partly contribute to the adipocyte's ability to protect ALL cells from DNR.

### Exogenous antioxidants protect ALL cells from DNR

**Figure 6:**
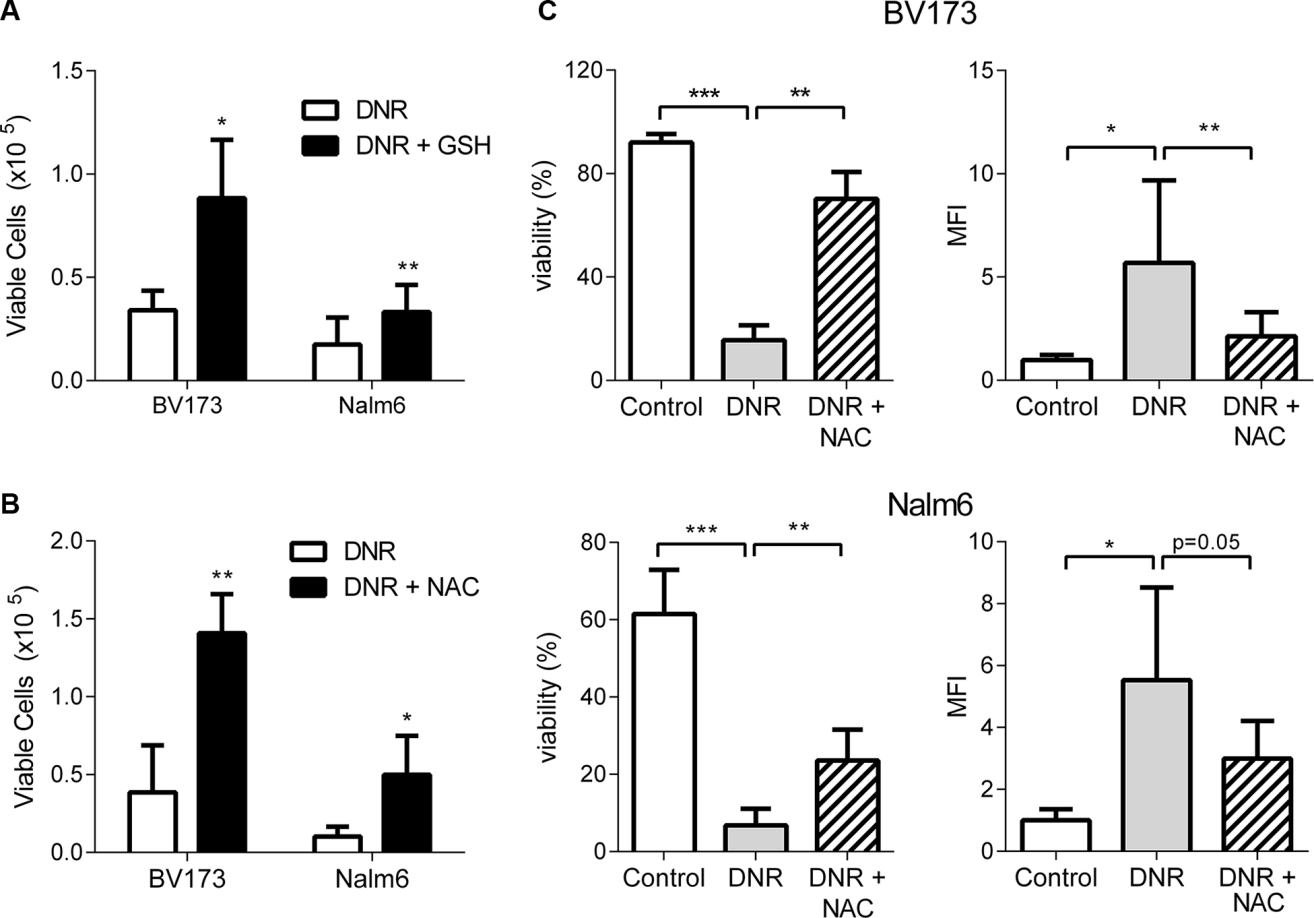
Exogenous antioxidants protect ALL cells from DNR (**A**) BV173 and Nalm6 treated with DNR or DNR + GSH (20 mM, *n* = 4). (**B**) BV173 and Nalm6 treated with DNR or DNR + NAC (20 mM, *n* = 3–4). (**C**) Cell viability and intracellular ROS evaluation using flow cytometry with DAPI and CellROX^®^ staining of BV173 (top two panels) and Nalm6 (bottom two panels) treated with DNR alone or DNR and NAC *n* = 5. *P < 0.05, **P < 0.01, ***P < 0.001 All asterisks indicate comparison to control conditions unless otherwise indicated.

As a proof of principle, we next tested whether reduction of ALL cell oxidative stress alone could protect ALL cells against DNR. Supplementation with high concentration (20 mM) of GSH significantly increased the number of viable cells after DNR treatment of BV173, Nalm6 (Figure 6A), and RS4;11 (data not shown) ALL cells. N-acetylcysteine (NAC), a GSH precursor, also protected these cells from DNR (Figure 6B), and was associated with increased viability assessed by flow cytometry (Figure 6C). NAC also partially reversed the induction of intracellular ROS caused by DNR treatment, measured by CellROX^®^, though this did not reach statistical significance in Nalm6 cells (Figure 6C).

## DISCUSSION

In the present study, we demonstrate that ALL cells induce oxidative stress in adipocytes, which causes them to protect the ALL cells from oxidative stress, in turn promoting resistance to the anthracycline, daunorubicin (DNR). These findings add to the growing literature which implicates adipocytes as an active and important component of the microenvironment of some cancers, such as breast cancer, ovarian cancer, and chronic lymphocytic leukemia [3–6, 29]. Since adipocytes are highly prevalent in the microenvironments of these cancers (breast, peritoneum, bone marrow), this phenomenon could contribute to cancer cell resistance to chemotherapy in patients.

Our results have uncovered a two-way communication between ALL cells and adipocytes, apparently initiated by ALL cell induction of oxidative stress in adipocytes. There is an expanding literature showing that cancer cells can alter the phenotypes of cells in their microenvironment, for example by inducing the development of “cancer-associated fibroblasts” [30–32]. Some of this interaction may be mediated by tumor cell induction of oxidative stress in nearby stromal cells [33]. Less is known about adipocytes in the cancer microenvironment, though recent studies have described “cancer-associated adipocytes”, which exhibit a modified phenotype and specific biological features [34], such as delipidation, dedifferentiation, and secretion of specific adipokines [35, 36]. These effects may be mediated by soluble factors released by tumor cells [37, 38]. Ours is the first study to our knowledge to identify this phenomenon between adipocytes and ALL cells.

Our findings are consistent with other literature which highlights the importance of oxidative stress to leukemia cell drug response. Inhibition of the oxidative stress response has been shown to be cytotoxic and enhance chemosensitivity of some leukemia cells [39–41]. Bone marrow stromal cells support primary ALL cell growth *in vitro* by providing cysteine, which maintains glutathione levels and results in protection against oxidative stress [42]. It is possible that adipocytes in bone marrow and adipose tissue provide similar protection.

While these data suggest that soluble factors mediate adipocyte protection of ALL cells from oxidative stress, we have yet to identify which factors are responsible. Among the top 10 genes upregulated in adipocytes (Table 1), adrenomedullin (ADM) is a cytokine known to be secreted by adipocytes [43], and has been shown to play a role in cell survival in several types of cancer [44]. In our hands, ADM did not protect 8093 ALL cells from DNR treatment (Supplementary Figure S1A), and neutralizing ADM antibodies did not block adipocyte protection of 8093 cells against DNR (Supplementary Figure S1B). These results excluded ADM as the relevant survival factor in our experimental system. Other adipocyte-secreted factors, such as MMP9 and IL8, have been shown to have tumor-promoting effects [4]. Therefore, further investigation is needed to identify which specific factors are responsible for adipocyte protection of ALL cells. Another weakness in the current study is that these results have not been confirmed in primary human ALL cells, nor *in vivo*. However, the use of multiple human ALL cell lines, coupled with our previous results demonstrating the effects of adipose tissue *ex vivo* [13], supports the potential importance of these phenomenon to human ALL.

Along with some of our previous studies [13, 16], this work highlights the important role of adipocytes in ALL progression and drug resistance. While adipocyte protection of ALL cells from oxidative stress is one potential mechanisms explaining the association between obesity and poor ALL prognosis [7, 9], there are likely others. Indeed, we have shown that adipocytes protect ALL from multiple chemotherapies [16], some of which do not act through induction of oxidative stress. In addition, we have found that adipocytes absorb some chemotherapies [45], including DNR [46], which could contribute to some of the present results, though would not explain the protection observed from adipocyte conditioned media. Thus, adipocyte protection from oxidative stress should be considered when developing therapeutic strategies which target oxidative stress in cancer cells [41].

## MATERIALS AND METHODS

### Chemicals and reagents

Daunorubicin (DNR), chaetocin (CTN), buthionine sulfoxine (BSO), auranofin (AUR), and other chemicals were obtained from Sigma Aldrich. Cell culture media components, DCFH-DA (dichloro-dihydro-fluorescein diacetate, a fluorimetric probe for oxidative stress assessment), and CellROX^®^ reagent were obtained from Life Technologies. Fetal bovine serum (FBS) was from Denville Scientific. Caspase 3, HIF-1α, and GAPDH antibodies were purchased from Cell Signaling Technologies. HO-1 antibodies were purchased from Abcam.

### Cell culture

All human ALL cell lines (BV173, RS4;11, and Nalm6) and murine pre-adipocyte 3T3-L1 were from ATCC. Murine pre-B 8093 ALL cells have been previously described [16]. Chub-S7 (immortalized human pre-adipose cell line) was from Nestec LTD and cultured as previously described [47, 48].

All human ALL cell lines were cultured in RPMI 1640 (Invitrogen), supplemented with 10% fetal bovine serum (FBS), sodium pyruvate (1 mM), Glutamax (2 mM), and gentamicin (10 μg/mL). 8093 cells were cultured in McCoy's 5A (Invitrogen), supplemented as above. Fresh recombinant murine interleukin-3 (Peprotech) and beta-mercaptoethanol (Sigma Aldrich) were added to 8093 cells at each passage. 3T3-L1 cells were cultured in DMEM high glucose (Invitrogen) supplemented with 10% FBS, sodium pyruvate (1 mM), Glutamax (2 mM), and gentamicin (10 μg/mL). Chub-S7 cells were cultured in DMEM/F12 (Invitrogen) with 10% FBS, sodium pyruvate (1 mM), Glutamax (2 mM), and gentamicin (10 μg/mL). 3T3-L1 (murine) and ChubS7 (human) pre-adipocytes were differentiated into adipocytes as previously described [48].

Co-culture experiments were performed by seeding ALL cells in chambers of polycarbonate 0.4 μm pore size TransWells (Corning Inc., Corning, NY). The TransWells were inserted in plates containing pre-adipocytes or adipocytes, such that the ALL cells were not in physical contact with the feeder cells.

Conditioned media were made with RPMI1640 containing 10% FBS. Leukemia cell conditioned media (LCM) were made by seeding 200,000 BV173 cells per mL in culture for 48 hours. Adipocyte conditioned media (ACM) were made by conditioning 100 μL/cm^2^ for 48 hours. Adipocyte leukemia cell conditioned media (ALCM) were made by seeding 200,000 per mL of BV173 in co-culture with a monolayer of adipocytes at 100 μL/cm^2^. All media were filtered upon collection using 0.22 μm syringe filters (Millipore) and stored at −20°C until use, avoiding freeze-thaw cycles.

### Gene expression profiling

3T3-L1 adipocytes were cultured in either LCM or regular media for 24 hours in biological triplicates. RNA was extracted and purified using Qiazol reagent and RNEasy Mini Kits as per the manufacturer's (Qiagen) instructions. Microarray hybridization was performed by the Genome Core facility at the Saban Research Institute of Children's Hospital Los Angeles (CHLA). RNA quality was first assessed using an Agilent Bioanalyzer (Agilent Technologies). RNA was then converted to cDNA with Superscript Choice for cDNA Synthesis (Invitrogen) and then converted to biotinylated cDNA using an Enzo High Yield RNA Transcript labeling kit (Enzo Diagnostics). GeneChip^®^ Mouse Gene ST Array (Affymetrix) was used for hybridization of cDNA. Genes that showed significant up/down-regulation (*p* < 0.05 compared to control conditions) in LCM treated samples were analyzed using Ingenuity Pathway Analysis (Ingenuity^®^ Systems, www.ingenuity.com).

### Real-time PCR

Cells were collected and stored with RNAProtect (Qiagen) and lyzed with QIAzol (Qiagen). RNA was extracted and purified with RNEasy Mini Kits (Qiagen). The quantity and quality of extracted RNA was measured by NanoVue (Denville Scientific). Two thousand nanograms of RNA from each condition were reverse transcribed to cDNA with a High Capacity 1st Strand Synthesis kit (Applied Biosystems). The expression of selected genes was quantified by qPCR using 25 ng of cDNA, Power SYBR Select PCR Master Mix (Applied Biosystems), and 200 nM primers designed using National Center for Biotechnology Information Primer-BLAST (See Supplementary Table S1 for primer sequences). Gene expression levels were quantified using the ABI 7900HT Sequence Detection System with the following thermal profile: 10 minutes at 95°C followed by 40 repeats of 95°C for 15 seconds, 60 degrees for 1 minute, and a final dissociation stage of 95°C for 15 seconds, 60°C for 15 seconds, and 95°C for 15 seconds. Transcript levels were normalized to β-actin. Fold change was calculated using the ΔΔCt method.

### Confocal microscopy

The intracellular ROS assay was done by incubating cells with 10 μM DCFH-DA for 15 minutes at 37°C and then washing with cold PBS. Samples were imaged at the Cellular Imaging Core at the Saban Research Institute of CHLA. Briefly, DCF fluorescence images were acquired with an LSM 700 confocal system mounted on an AxioObserver.Z1 microscope equipped with a 63×/1.4 Plan-APOCHROMAT objective lens and controlled with ZEN 2009 software (Carl Zeiss Microscopy, Thornwood, NY). A 488 nm laser and 560 nm long-pass filter were used for fluorescence excitation and emission. Transmitted laser light was collected to form a DIC image simultaneously with the fluorescence image. Images were histogram-stretched in a consistent manner to increase brightness for publication. Intracellular ROS signals from the images were quantified using ImageJ (National Institute of Health, Bethesda, MD, USA).

### Western blots

Total protein was extracted from cells using protein isolation buffer (62.5 mL Tris-HCL, 2% w/v SDS, 1% v/v Igepal CA-630, 10% glycerol, 0.01 mg/mL aprotinin, 1 mM phenylmethanesulphonylfluoride, and Phosphatase Inhibitor Cocktail Set II (Calcbiochem)). Lysates were sonicated briefly and centrifuged for 10 minutes at 15,000 g at 4°C. The supernatant was retained and protein concentration was quantified by BCA assay (Pierce Biotechnology). Equal amounts of protein was subjected to SDS-PAGE and transferred to a nitrocellulose membrane. Membranes were blocked in 5% milk and then probed with specific primary antibodies. The secondary antibodies used were HRP-linked. Bands were detected using a HyGLO-HRP detection kit (Denville Scientific, South Plainfield, NJ, USA) and developed with HyBLOT-CL Autoradiography Film (Denville Scientific). Densitometric band analysis was performed using ImageJ.

### Glutathione measurements

The GSH-Glo^™^ Glutathione Assay (Promega, Madison, WI) was used to measure glutathione (GSH) in media and on cell extracts, following the manufacturer's instructions. The protein amount of each sample was quantified by BCA assay for normalization of GSH.

### Flow cytometry

ALL cells were treated with 10 μM DCFH-DA for 15 minutes at 37°C, then the reaction was stopped by transferring the cells on ice and protecting from light. Then DAPI was added to distinguish live cells. The samples were run at the Fluorescence Activated Cell Sorting Core at CHLA on LSR II Analyzer from Becton Dickinson (BD). The PE channel was used to detect intracellular DNR and the FITC channel for DCF. Proper compensation controls were used to calculate spill over between PE and FITC channels. In other experiments where CellROX^®^ Deep Red Reagent (Life Technologies) was used, the APC channel was used to detect CellROX^®^. For apoptosis analysis, ALL cells were harvested and stained with 25 ng APC-conjugated Annexin V for 15 minutes at room temperature. Then 50 ng DAPI was added to stain for late apoptotic/necrotic cells. The samples were run on the same analyzer above.

### Statistical analysis

All statistical tests were performed with Microsoft Excel 2010. The data are presented as mean ± Standard Deviation. Experimental groups were compared using paired, two-sided *t*-tests; non-normally distributed data was log-transformed prior to *t*-test. A *p* value of less than 0.05 was taken as statistically significant. Flow cytometry of intracellular DNR and DCF were quantified using median fluorescence intensity (MFI). MFI was calculated by dividing sample fluorescence signals by the control signal. All experiments were performed in triplicates and repeated at least three times. All graphs are presented as mean ± SD.

## SUPPLEMENTARY MATERIALS TABLE FIGURES


