# Supplementary material for: Adipocytes cause leukemia cell resistance to daunorubicin via oxidative stress response
Source: Oncotarget. 2016 Sep 26;7(45):73147–59. doi: 10.18632/oncotarget.12246 (PMC5341969; doi:10.18632/oncotarget.12246)
Supplement: Supplementary file 1 [file oncotarget-07-73147-s001.pdf]

Adipocytes cause leukemia cell resistance to daunorubicin via oxidative stress response

Supplementary Materials

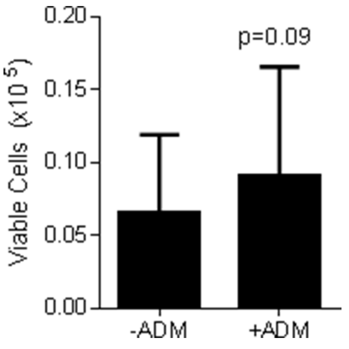

Supplementary Figure S1: ADM does not significantly protect 8093 cells from DNR (*n* = 5).

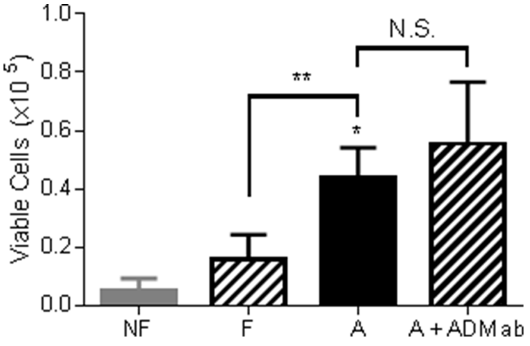

Supplementary Figure S2: ADM neutralizing antibody does not reverse adipocyte protection of 8093 against DNR (*n* = 3).

Supplementary Table S1: Primer list

Gene	Species	Forward	Reverse
β-actin	murine	5′-TTGCTGACAGGATGCAGAAG-3′	5′-AAGGGTGTAAAACGCAGCTC-3′
GAPDH	murine	5′-ACCACAGTCCATGCCATCAC-3′	5′-CACCACCCTGTTGCTGTAGCC-3′
GCLC	murine	5′-ACTGAATGGAGGCGATGTTC-3′	5′-AGTGATGGTGCAGAGAGCCT-3′
GCLM	murine	5′-TCCTTGGAGCATTTACAGCC-3′	5′-AGAGCAGTTCTTTCGGGTCA-3′
HO-1	murine	5′-CACGCATATACCCGCTACCT-3′	5′-CCAGAGTGTTCATTCGAGCA-3′
Mt-2	murine	5′-CCGCGTGCTTCTCTCCAT-3′	5′-ATCGACGAGAGATCGGTTTGA-3′
ADM	murine	5′-CTCGCTGATGAGACGACAGTTC-3′	5′-CTCTGGCGGTAGCGTTTGAC-3′
β-actin	human	5′-ACAGAGCCTCGCCTTTGCCG-3′	5′-CGATGCCGTGCTCGATGGGG-3′
GCLC	human	5′-AAACCCAAACCATCCTACCC-3′	5′-CGAGGGTGCTTGTTTATTGC-3′
GCLM	human	5′-TCAACCCAGATTTGGTCAGG-3′	5′-AGGCTGTAAATGCTCCAAGG-3′
